# An mHealth Intervention for Gay and Bisexual Men’s Mental, Behavioral, and Sexual Health in a High-Stigma, Low-Resource Context (Project Comunică): Protocol for a Randomized Controlled Trial

**DOI:** 10.2196/52853

**Published:** 2024-05-06

**Authors:** Corina Leluțiu-Weinberger, Mircea L Filimon, Donald Hoover, Mihai Lixandru, Lucian Hanu, Bogdan Dogaru, Tudor Kovacs, Cristina Fierbințeanu, Florentina Ionescu, Monica Manu, Alexandra Mariș, Elena Pană, Cristian Dorobănțescu, Adrian Streinu-Cercel, John E Pachankis

**Affiliations:** 1 School of Nursing Columbia University New York, NY United States; 2 Department of Statistics Rutgers, the State University of New Jersey Piscataway, NJ United States; 3 The Romanian Association Against AIDS Bucharest Romania; 4 MozaiQ Bucharest Romania; 5 Insight Bucharest Romania; 6 Mariș Alexandra - Cabinet Individual de Psihologie Bucharest Romania; 7 Obregia Hospital Bucharest Romania; 8 Data Center Solutions Bucharest Romania; 9 The National Institute of Infectious Diseases “Professor Dr. Matei Balș” Bucharest Romania; 10 Department of Social and Behavioral Sciences Yale School of Public Health New Haven, CT United States

**Keywords:** gay and bisexual men, HIV prevention, heavy alcohol use, stigma, mental health, behavioral intervention, mobile phone

## Abstract

**Background:**

The World Health Organization reported that 80% of new HIV diagnoses in Europe in 2014 occurred in Central and Eastern Europe. Romania has a particularly high HIV incidence, AIDS prevalence, and number of related deaths. HIV incidence in Romania is largely attributed to sexual contact among gay and bisexual men. However, homophobic stigma in Romania serves as a risk factor for HIV infection for gay and bisexual men. The Comunică intervention aims to provide a much-needed HIV risk reduction strategy, and it entails the delivery of motivational interviewing and cognitive behavioral therapy skills across 8 live text-based counseling sessions on a mobile platform to gay and bisexual men at risk of HIV. The intervention is based on the information-motivation-behavior and minority stress models. There is preliminary evidence suggesting that Comunică holds promise for reducing gay and bisexual men’s co-occurring sexual (eg, HIV transmission risk behavior), behavioral (eg, heavy alcohol use), and mental (eg, depression) health risks in Romania.

**Objective:**

This paper describes the protocol for a randomized controlled trial designed to test the efficacy of Comunică in a national trial.

**Methods:**

To test Comunică’s efficacy, 305 gay and bisexual men were randomized to receive Comunică or a content-matched education attention control condition. The control condition consisted of 8 time-matched educational modules that present information regarding gay and bisexual men’s identity development, information about HIV transmission and prevention, the importance of HIV and sexually transmitted infection testing and treatment, heavy alcohol use and its associations with HIV transmission risk behavior, sexual health communication, finding social support, and creating sexual health goals. Participants undergo rapid HIV and syphilis testing and 3-site chlamydia and gonorrhea testing at baseline and the 12-month follow-up. Outcomes are measured before the intervention (baseline) and at the 4-, 8-, and 12-month follow-ups.

**Results:**

The study was funded in September 2018, and data collection began in May 2019. The last participant follow-up was in January 2024. Currently, the data analyst is cleaning data sets in preparation for data analyses, which are scheduled to begin in April 2024. Data analysis meetings are scheduled regularly to establish timelines and examine the results as analyses are gradually being conducted. Upon completion, a list of manuscripts will be reviewed and prioritized, and the team will begin preparing them for publication.

**Conclusions:**

This study is the first to test the efficacy of an intervention with the potential to simultaneously support the sexual, behavioral, and mental health of gay and bisexual men in Central and Eastern Europe using motivational interviewing support and sensitivity to the high-stigma context of the region. If efficacious, Comunică presents a scalable platform to provide support to gay and bisexual men living in Romania and similar high-stigma, low-resource countries.

**Trial Registration:**

ClinicalTrials.gov NCT03912753; https://clinicaltrials.gov/study/NCT03912753

**International Registered Report Identifier (IRRID):**

DERR1-10.2196/52853

## Introduction

### Background

The World Health Organization reported that 80% of new HIV diagnoses in Europe in 2014 occurred in Central and Eastern Europe (CEE) [1]. Of the 15 surveyed European countries, Romania had the second-highest HIV incidence and AIDS prevalence and the highest number of AIDS-related deaths between 2005 and 2014 [1]. After the first wave of the HIV epidemic in Romania, which occurred in children via nosocomial infection in the 1980s [2-6], a new wave, largely attributed to male-to-male sexual contact, has emerged and is increasing [1,7,8]. The increased exposure of gay and bisexual men to HIV and other sexually transmitted infections (STIs) in the past 2 decades has purportedly resulted from more frequent travel to and from areas of high HIV prevalence outside Romania, including many European Union (EU) states in Western Europe [9]; lack of sexual education in Romania in general [10-12] and specifically sexual education that responds to gay and bisexual men’s distinct sexual health needs [13]; and pervasive stigma against gay and bisexual men [14].

Despite these co-occurring threats resulting in increased HIV transmission risk, gay and bisexual men remain underprioritized in Romanian public health [2]. Furthermore, reporting of epidemiologic data might be unreliable in Romania due to ineffective surveillance systems and gay and bisexual men’s anticipated stigma and normative identity concealment [15]. As a result, HIV transmission among gay and bisexual men is frequently underreported or misclassified as heterosexual [1,16,17]. For instance, an independent European survey found that the number of HIV diagnoses self-reported by Romanian gay and bisexual men respondents was 2.7 times the official national notification rate [17], suggesting that a significant proportion of HIV infections occurring among the gay and bisexual men community might be attributed to heterosexual transmission, thus yielding inaccurate guidance for national HIV-related priorities.

The best available evidence suggests that HIV prevalence among Romanian gay and bisexual men increased from <10% in 2009 to approximately 20% in 2014 [18-20]. An international biobehavioral survey of gay and bisexual men found that Romania had the highest rate of unrecognized HIV infections across all included European countries [19-22]. Untreated STIs represent a primary risk factor for HIV transmission [23], yet gay and bisexual men–sensitive screening for STIs is rare in Romania. For example, of 629 gay and bisexual men who sought an STI test over 1 year in the capital city of Bucharest, only 6.4% received both an anal swab and penile examination (compared to 72.4% in Amsterdam)—the lowest rate in all 40 cities participating in a European survey of gay and bisexual men (N=174,209). Furthermore, fewer than one-third of Romanian gay and bisexual men had been screened for STIs in the previous year, and fewer than half knew where they could access STI testing [20,24].

Insufficient sexual health knowledge specific to gay and bisexual men in Romania has direct implications for HIV transmission. A 2017 survey of 50 European and contiguous countries found that, of 2002 Romanian gay and bisexual men respondents, only 0.4% had ever used pre-exposure prophylaxis (PrEP) and 1.1% had ever used postexposure prophylaxis, whereas 62% were unaware of PrEP and only 56% were knowledgeable regarding *undetectable=untransmissible*. A total of 13% of respondents reported having at least 2 steady sex partners in the previous year with whom they did not use condoms (the third highest rate among the 50 surveyed countries); 32% reported having condomless sex due to lack of access to condoms (the seventh highest rate among the 50 surveyed countries) [25]. Of those who had never been tested for HIV (approximately 50% in the Romanian sample), half (51%) did not know where to get tested (the fifth highest rate among the 50 countries) [25].

In terms of mental and behavioral health needs that can co-occur with HIV transmission risk among gay and bisexual men [26,27], European surveys indicate that 12% of Romanian gay and bisexual men reported severe anxiety and depression during the previous 2 weeks, 21% had thoughts of self-harm [25], and 14% met criteria for alcohol dependency. Homophobic stigma is also a risk factor for HIV transmission and co-occurring mental and behavioral health risks as it keeps most Romanian gay and bisexual men hidden and out of reach of official HIV and STI surveillance and the few available prevention services. Depression, anxiety, heavy alcohol use, and homophobic stigma combine to create a syndemic affecting gay and bisexual men [28-30].

Despite gains in some rights for lesbian, gay, bisexual, transgender, and queer (LGBTQ) individuals (eg, decriminalization of homosexuality) over the past 2 decades, Romania remains one of the most structurally homophobic countries in Europe [31,32], with structural homophobia being associated with low life satisfaction, poor mental health, social isolation, internalized homonegativity, and high degrees of identity concealment among gay and bisexual men [15,31,33]. Identifying as an ethnic minority group (eg, Roma) may further amplify the odds of encountering hate speech and harassment [34], and there might be intersectional challenges for ethnic minority gay and bisexual men in Romania [35]. A recent report by the EU Agency for Fundamental Rights found significant physical or sexual attacks against LGBTQ people in Romania (15%) [34]. Of 28 EU member states, Romania was among the top 3 countries in terms of the prevalence of hate-motivated physical or sexual attacks against LGBTQ people [34]. Perhaps as a further manifestation of structural stigma toward gay and bisexual men, no governmental funds are currently allocated for HIV and STI prevention among gay and bisexual men in Romania despite the clear and increasing need for such prevention, as outlined previously [2,18,22,36].

Evidence-based HIV prevention interventions for gay and bisexual men are not widely available in Romania and are rare in the CEE region, where most HIV-related interventions have been developed for people who inject drugs [37]. A systematic review in 2015 identified only 24 HIV prevention interventions for gay and bisexual men in Europe [38], none of which appeared to have been implemented in Romania. To address this gap and respond to the interlocking challenges facing gay and bisexual men in Romania that contribute to their increasing HIV transmission risk behavior, our team sought to adapt a promising intervention created for gay and bisexual men in the United States [39], which was recently pilot-tested in Romania, under the name of Comunică, to support this population’s unmet needs of [40].

### Comunică Intervention Background and Pilot

Comunică is based on the information–motivation–behavioral skills (IMB) model of health behavior change [41], which postulates that individuals must possess the requisite information for enacting sexual health; motivation to address their HIV transmission risk behavior, alcohol use, and mental health; and behavioral skills necessary for reducing risk behaviors. The Comunică intervention is guided by motivational interviewing (MI) principles and techniques [42] to provide accurate information about HIV transmission, heavy alcohol use, and local gay and bisexual men–affirmative health resources (eg, HIV-testing sites) and build motivation to improve behavioral skills via cognitive behavioral skill training (CBST) [43]. MI is an evidence-based form of person-centered therapeutic communication that privileges client values and preferences for change to help individuals resolve ambivalence and move toward their valued goals [41,44]. For individuals who attain a high degree of motivation for change [45], CBST is used to promote awareness of contextual triggers and unhealthy behavioral patterns and teach coping skills to reduce personal risk [46]. Given the stigmatizing context of Romania, the CBST skills in Comunică are presented in a manner that acknowledges the barriers posed by social stigma to gay and bisexual men’s health while empowering them to circumvent these barriers by building self-efficacy, learning effective communication, and implementing planful problem-solving [47-49]. In this way, Comunică also draws on minority stress theory recognizing that stigmatizing societal contexts represent the ultimate source of health disparities affecting gay and bisexual men [50]. Minority stress content informs the context in which the aforementioned psychoeducational information is presented, such as through a focus on identity development in the context of stigma and the ways in which minority stress affects mental, behavioral, and sexual health.

The Comunică intervention is delivered across 8 sessions by a trained counselor via synchronous (ie, live) text-based chat on a mobile-optimized website that also contains features for weekly tracking of HIV transmission risk behavior, heavy drinking, and mood. The study platform allows gay and bisexual men randomized to the Comunică intervention to track their weekly number of condomless sex acts, number of partners, number of heavy alcohol use days, and positive and negative moods [51]. A review of this information with counselors during sessions is intended to support motivation and create contextually informed behavior change goals. Previous research has shown that mobile tools are the primary means for Romanian gay and bisexual men to form and navigate social and sexual networks, especially given normative identity concealment [31], offering an ideal intervention platform [40]. While session content is driven by participant-selected priorities, they are discussed within the general theoretical and counseling frameworks of the Comunică intervention.

The feasibility, acceptability, and preliminary efficacy of Comunică was established between 2014-2016 in an open-trial pilot study with 43 young gay and bisexual men in Romania (mean age 23.2, SD 3.6; range 17-29 years) who reported condomless anal sex (CAS) acts with a male partner and at least 5 days of heavy drinking (≥5 drinks on one occasion) in the previous 3 months [40]. Specifically, gay and bisexual men who received Comunică reported, from baseline to a 3-month follow-up, significantly reduced depression, anxiety, and heavy alcohol use (*P*=.005) and increased condom use self-efficacy (*P*=.01), HIV-related knowledge (*P*=.001), and HIV testing (*P*=.05) [40]. While reductions in CAS trended in the expected direction from baseline to postintervention follow-up (mean 14.7 vs mean 10.8), the analyses were not sufficiently powered to detect significant differences. The Comunică intervention was created through in-depth consultation with 22 Romanian gay and bisexual men and 6 community stakeholders (eg, gay and bisexual men advocates and service providers) and was based on a chat-based MI intervention established in the United States [39].

### Study Objectives

This study aims to test the efficacy of Comunică in reducing HIV transmission risk behavior (ie, number of CAS acts with HIV-positive or unknown-status partners outside the context of one’s own or one’s primary partner’s adherent PrEP use or undetectable viral load in the past 30 days) in a randomized controlled trial of 305 Romanian gay and bisexual men. The secondary outcomes include depression, heavy alcohol use, and HIV and STI testing. The control condition consists of 8 time-matched educational modules that present information regarding gay and bisexual men identity development, HIV and STI prevention, heavy alcohol use and its associations with HIV transmission risk behavior, sexual health communication, and the importance of social support created in consultation with Romanian gay and bisexual men community members and advocates. The educational modules for the control condition are hosted on the same study website as the Comunică intervention but without access to counseling or behavioral and mood-tracking features.

This study also tests two sets of mechanisms of intervention efficacy: (1) motivational mechanisms derived from the IMB model [41,44,52] (eg, knowledge of sexual health and alcohol use effects, motivation to reduce HIV risk and heavy alcohol use, and self-efficacy for safer sex and reductions in alcohol use) and (2) minority stress mechanisms derived from minority stress theory (eg, rejection sensitivity, stigma consciousness, and identity concealment) [53]. If shown to be efficacious in this trial, Comunică will constitute a unique mental, behavioral, and sexual health intervention that can be implemented in Romania and other high-stigma, low-resource national contexts in the future.

## Methods

### Design

**Figure 1:**
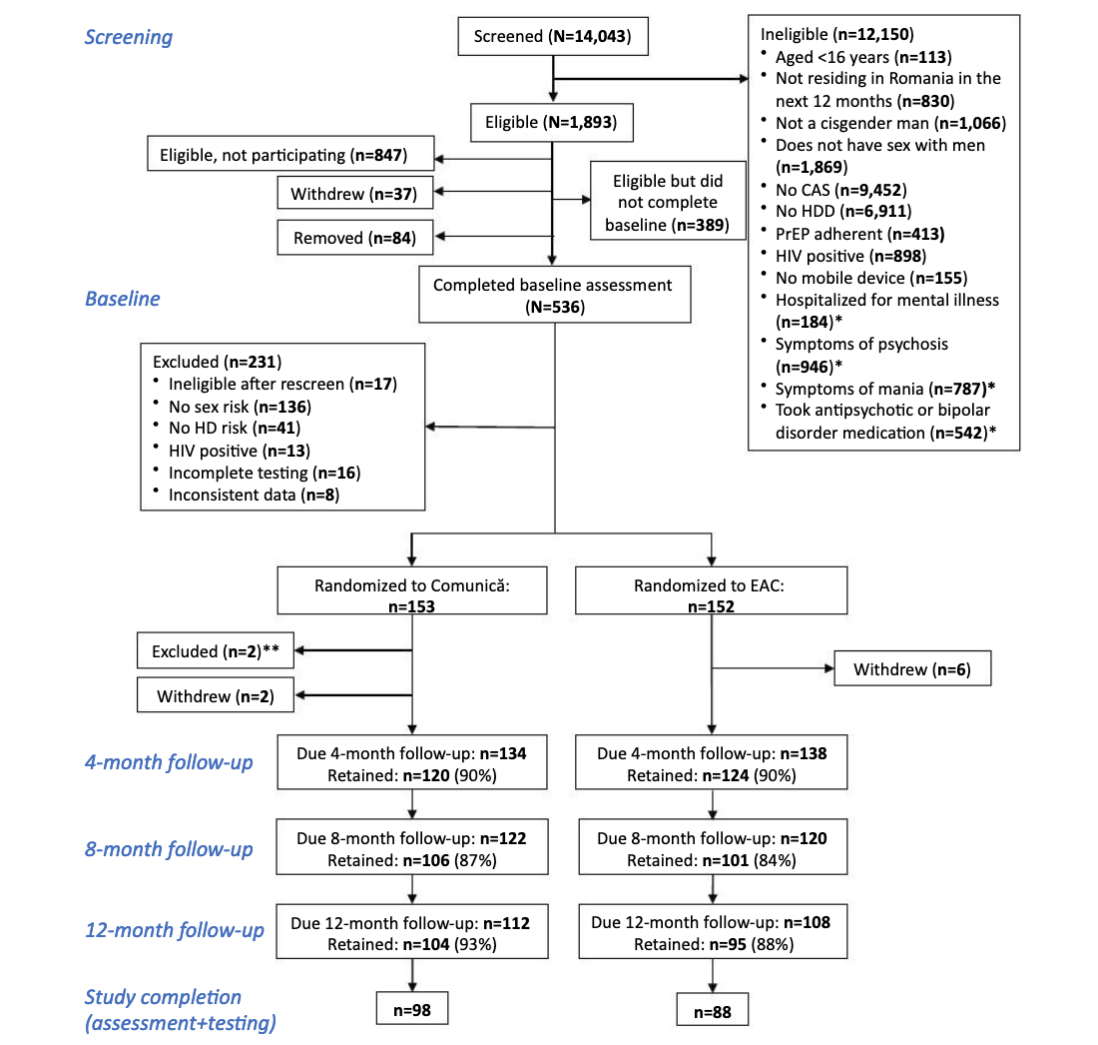
CONSORT (Consolidated Standards of Reporting Trials) diagram. *As of September 16, 2020, mental health screening questions are no longer part of eligibility criteria. **These participants admitted to providing false condomless anal sex (CAS) or heavy drinking days (HDD) data at baseline assessment. EAC: education attention control; HD: heavy drinking; PrEP: pre-exposure prophylaxis.

In this randomized controlled trial, Comunică is compared to a content-matched education attention control (EAC) condition in changing (1) the primary outcome (frequency of CAS acts with HIV-positive or unknown-status partners outside the context of one’s own or one’s primary partner’s adherent PrEP use or viral suppression) and (2) secondary outcomes (depression, anxiety, suicidal thoughts, heavy alcohol use, and HIV and STI testing). Both the intervention and control groups received 8 one-hour intervention sessions or educational modules, respectively, to be completed over the course of 4 months. Outcomes are measured before the intervention (baseline) and at the 4-, 8-, and 12-month follow-ups. All participants self-administer at-home rapid testing for HIV and syphilis, and self-collect sampling (urethral, pharyngeal, and rectal) for chlamydia and gonorrhea sent to a laboratory at baseline and the 12-month follow-up. The CONSORT (Consolidated Standards of Reporting Trials) diagram in Figure 1 outlines the flow of participants throughout the study as of September 12, 2023.

### Recruitment

Recruitment and enrollment for the study ended in January 2023, with follow-up completion in January 2024. Gay and bisexual men living in or within 40 miles of 10 cities (ie, București, Brașov, Timișoara, Cluj-Napoca, Iași, Constanța, Suceava, Craiova, Galați, and Satu Mare) were recruited and screened. By selecting these cities, this study covers all regions of the country [54]. Collaborating LGBTQ advocacy organizations posted study advertisements on their websites, subscriber lists, and key virtual venues (eg, Grindr, Facebook, and Instagram). Study recruitment also relied on word of mouth; for instance, recruiters approached men in gay and bisexual men–prevalent venues (bars, events, and public cruising areas). Finally, enrolled participants were encouraged to share study information with their peers. Nevertheless, 44.9% (137/305) of participants were recruited on Grindr, and 41% (125/305) were recruited on Facebook.

### Eligibility

#### Inclusion Criteria

Gay and bisexual men were eligible if they reported (1) male sex at birth and current male identity, (2) age of ≥16 years, (3) ≥1 act of CAS with an HIV-positive or status-unknown male partner in the previous 30 days, (4) ≥1 heavy drinking day in the previous 30 days (ie, ≥5 standard alcoholic drinks on 1 occasion per month [55,56]), (5) owning a mobile device (smartphone, tablet, or laptop), (6) residence in Romania for the duration of study participation (12 months), and (7) nonadherence to PrEP and (8) were confirmed to be HIV negative upon testing at baseline.

#### Exclusion Criteria

Participants were excluded if they demonstrated active suicidality, psychosis, or mania.

### Screening

Potential participants completed an eligibility screener on Qualtrics (Qualtrics International Inc), a research-designated secure Health Insurance Portability and Accountability Act–compliant web-based software [57]. Eligible gay and bisexual men had the option to provide contact information at the end of the screener in a separate survey for research staff to later contact them to review the study in more detail and assess consent.

### Consent

A research staff member contacted eligible participants via phone to review the consent form and verify their age. Points of confusion were clarified, and individuals still interested in participating submitted an electronic consent form via Qualtrics [57]. The research staff outlined the steps to take place immediately following consent, namely, that participants would receive a link to the baseline assessment and a numerical study identification number to be used for the duration of the study for confidentiality purposes, undergo HIV and STI testing, and be randomized to one of the study conditions. Participants were able to use any mobile device (smartphone, tablet, or laptop) to complete the sessions.

### Randomization

After baseline, participants were randomized to the Comunică or EAC condition based on a list of random numbers generated by the study biostatistician. A blocked stratified randomization scheme was used, with 150 participants each being randomized 1:1 to each arm within the following four strata based on past 3-month self-reported baseline number of HIV transmission risk behavior sex acts and heavy alcohol use days as follows: (1) lower CAS acts (≤2 acts) and lower alcohol use (≤5 drinks); (2) lower CAS acts (≤2 acts) and higher alcohol use (>5 drinks); (3) higher CAS acts (>2 acts) and lower alcohol use (≤5 drinks); and (4) higher CAS acts (>2 acts) and higher alcohol use (>5 drinks). The 150 persons within each stratum were randomized using 15 blocks of size 4 and 15 blocks of size 6, with the order of the 30 total blocks being randomly permuted.

Counselors contacted participants assigned to the Comunică condition to schedule the first session, at which point they both logged onto the study platform. Group assignment was not masked from study staff given that all assessments were self-administered.

### EAC Condition

The EAC condition consisted of 8 educational modules based on the team’s experience with HIV prevention education with gay and bisexual men in the United States and Romania [15,39,40,48,58,59] and included the following topics: (1) gay and bisexual men identity, (2) “HIV 101” (eg, transmission risks, prevention, and treatment), (3) the importance of HIV and STI testing and treatment, (4) alcohol and the body, (5) the role of alcohol in HIV transmission risk behavior, (6) HIV status disclosure and sexual health communication, (7) finding social supports and safety, and (8) creating and reaching sexual health goals. The content was finalized in collaboration with staff of Romanian LGBTQ-supportive organizations. To maximize participant engagement and learning, each module contained a 5-item quiz (with correct answers subsequently provided) and exercises and vignettes prompting participants to provide answers based on their understanding of the topics and their own experience.

To minimize contamination, counselors who administered Comunică did not interact with EAC participants, and Comunică and EAC materials were only accessible through unique log-in credentials known only to participants.

### Counselor Training and Fidelity Monitoring

In 2019, before trial commencement, 5 psychologists living in Romania completed a 2-day training on the intervention. Of these psychologists, 2 delivered the Comunică intervention in the pilot study [40], and 3 were recruited from a pool of 54 mental health professionals whom the principal investigators (PIs) had previously engaged in a separate study [60]. The training included didactic and experiential components; a review of MI, CBST, the IMB model, and minority stress theory; unique facets of delivering the intervention via text; and reviewing vignettes from the pilot study. The counselors practiced delivering each session on the intervention platform, taking turns being a mock participant and counselor, and receiving biweekly remote video supervision from a clinical supervisor. At the start of the trial, the team reviewed all session transcripts. Once intervention fidelity was attained, the team randomly selected slightly over half of the subsequent sessions to verify fidelity to the intervention content and adherence to MI principles and techniques. As in the pilot study, the counselors translated each session transcript into English for the clinical team’s review.

### Study Assessments

Participants provided assessment data at baseline and at 4-, 8-, and 12-month follow-up appointments, self-administered via Qualtrics [57] and a web-based platform designed specifically for this study to capture past–30-day sexual and alcohol use behaviors in a self-administered calendar review. Participants also completed biological testing for HIV, syphilis, gonorrhea, and chlamydia at the baseline and 12-month follow-up appointments. Finally, all retained participants completed an exit survey after their 12-month follow-up that assessed the acceptability of the intervention. Based on each completed portion of the study, participants were compensated in Romanian RON equivalent to US $20, US $25, US $30, and US $40 for the baseline, 4-, 8-, and 12-month assessments, respectively, and US $10 per session based on each completed portion of the study. The measures are described in the following sections.

### Demographics

At baseline, as shown in Table 1, participants indicated their age, sexual orientation, sex assigned at birth, current gender identification, income, residence (rural vs urban), ethnicity, and educational level. Participants also indicated the age at which they attained sexual orientation developmental milestones (eg, age of awareness of attraction to men) [61].

**Table 1:** Baseline characteristics of the enrolled participants (N=305).

Characteristic			Participants, n (%)
**Age (y)**			
	16-29	232 (76.1)	
	30-39	56 (18.4)	
	40-49	16 (5.2)	
	≥50	1 (0.3)	
**Sexual identity**			
	Gay	205 (67.2)	
	Bisexual	92 (30.2)	
	Queer	2 (0.7)	
	Pansexual	2 (0.7)	
	Uncertain	4 (1.3)	
**Educational level**			
	High school or lower	78 (25.6)	
	Vocational studies	9 (3)	
	Some college	108 (35.4)	
	College degree	57 (18.7)	
	Graduate degree	53 (17.4)	
**Relationship status**			
	Single and not dating	97 (31.8)	
	Dating	98 (32.1)	
	In a serious relationship	110 (36.1)	
**Ethnicity**			
	Romanian	279 (91.5)	
	Hungarian	22 (7.2)	
	Roma	3 (1)	
	Other	1 (0.3)	
**Employment**			
	Full time	149 (48.9)	
	Part time	18 (5.9)	
	Unemployed	15 (4.9)	
	Student	121 (39.7)	
	On disability	2 (0.7)	
**High school location**			
	Small town	161 (52.8)	
	Medium to large town or city	144 (47.2)	
**Age of awareness of attraction to men (y)**			
	4-14	230 (75.4)	
	15-25	72 (23.6)	
	26-34	3 (1)	

### Primary Outcome

This study’s primary outcome, HIV transmission risk behavior, is the frequency of CAS acts with HIV-positive or unknown-status partners outside the context of one’s own or one’s primary partner’s adherent PrEP use or undetectable viral load in the past 30 days. The partner’s adherence to PrEP use was verified by the participant having witnessed their partner taking their medication daily. The partner’s viral suppression in the past 30 days was verified by the participant having seen current test results or witnessing that partner taking antiretroviral therapy. To report past–30-day sexual behavior, participants completed a self-administered web-based Timeline Followback (TLFB) interview [62,63]. For each sexual act on each day, participants reported partner type (eg, primary or casual), partner gender, partner HIV status and known viral suppression (if applicable), type of sexual behavior (eg, insertive anal sex), condom use, PrEP use by themselves or their partner, and whether they were under the influence of alcohol during reported sex. TLFB collects retrospective day-level data and has been validated for electronic self-administration [62]. TLFB has good test-retest reliability, convergent validity, and agreement with collateral reports for sexual behavior [64,65] and alcohol use [66]. In this study, we developed a mobile-optimized web-based platform for the TLFB and iteratively improved it during a usability testing phase with local gay and bisexual men community members.

### Secondary Outcomes

#### Heavy Alcohol Use

TLFB also asks participants to report their past–30-day heavy alcohol use, including whether it took place before or during sex. Participants also complete the 3-item Alcohol Use Disorders Identification Test–Consumption scale (AUDIT-C) [67], a standardized measure of alcohol-related problems.

#### Mental Health

Participants complete the Center for Epidemiologic Studies Depression Scale (CES-D) as a measure of depression symptoms [68], the Beck Anxiety Inventory (BAI) as a measure of anxiety symptoms [69], and the Suicidal Ideation Attributes Scale (SIDAS) [70] as a measure of suicidal ideation.

### Potential Intervention Mediators

#### IMB Model

As informed by the IMB model, knowledge acquisition and motivation to reduce HIV transmission risk behavior and heavy alcohol use will be assessed as potential mechanisms of intervention efficacy. Information is measured using the Sexual Health Knowledge Questionnaire [71] and the Alcohol Attitudes Questionnaire [72]; motivation to reduce CAS and alcohol use is measured using the University of Rhode Island Change Assessment Scale [73] and the Stages of Change Readiness and Treatment Eagerness Scale [74], respectively; and behavioral self-efficacy to reduce HIV transmission risk behavior and heavy alcohol use is measured using the Safer Sex Efficacy Questionnaire [75] and Confidence in Reducing Alcohol Use Questionnaire [76], respectively.

#### Minority Stress Pathways

We measured potential mechanisms suggested by minority stress theory [77], including sexual orientation concealment using the concealment motivation subscale of the Lesbian, Gay, and Bisexual Identity Scale (LGBIS) [78]; rejection sensitivity using the acceptance concerns subscale of the LGBIS [78]; internalized stigma using the internalized homonegativity subscale of the LGBIS [78]; assertiveness using the Rathus Assertiveness Schedule [79]; and social support using the Multidimensional Scale of Perceived Social Support [80].

The protocol was also approved by the Bioethics Committee of the National Institute for Infectious Diseases the European Men-Who-Have-Sex-With-Men Internet Survey [21], participants indicate the frequency of their HIV or STI testing. Participants are asked whether they provided a blood sample to test for any STIs; whether their penis and anus were examined and swabbed as part of any STI testing in the previous 4 months; and whether and, if so, when (eg, past 7 days or 4 weeks) they were diagnosed with chlamydia, gonorrhea, genital warts, herpes, syphilis, hepatitis B or C, or urethritis.

### HIV and STI Testing, Counseling, and Linkage to Services

Participants complete HIV and STI testing after providing consent and completing the baseline and 12-month follow-up assessments. Specifically, a testing counselor affiliated with the study and based at a Romanian nongovernmental organization specialized in HIV and STI prevention and treatment for gay and bisexual men and other marginalized populations contacts the participant to provide two options: (1) mail an HIV and STI test kit to their home or (2) have the participant pick up the test at their offices. Participants receive a self-testing kit containing a rapid HIV and syphilis test and swabs and a urine collection container for pharyngeal, rectal, and urethral testing of chlamydia and gonorrhea. The testing counselor guides participants through the testing steps [81,82]. The HIV and syphilis test results are available within 20 minutes of testing. Participants are required to take a photograph of the results (marked with their study ID) and upload it to a study platform. The chlamydia and gonorrhea self-collected samples are mailed back to the organization, which mails them for analysis to a laboratory. Upon receiving results for chlamydia and gonorrhea testing (usually within 1 week of laboratory receipt), the testing counselor communicates the results to the participant. For positive or inconclusive test results, the testing counselor provides participants with the name and contact information of the study-affiliated infectious disease care provider in their area for confirmatory testing and treatment, as appropriate, and offers to assist with this linkage. Finally, participants are asked to complete a satisfaction survey at the 12-month follow-up [83].

### Ethical Considerations

#### Ethics Approval and Consent to Participate

This research was conducted in accordance with the Declaration of Helsinki, and all methods were carried out in accordance with relevant guidelines and regulations within the United States and Romania. This study was approved by the Columbia University Institutional Review Board (FWA0000263; protocol AAAU2518), Yale University Institutional Review Board (FWA00002571; protocol 2000024286), and Rutgers University Institutional Review Board (FWA00003913; protocol Pro2018000725). The protocol was also approved by the Bioethics Committee of the National Institute for Infectious Diseases “Prof. Dr. Matei Balș” in Bucharest, Romania (FWA00013199). All enrolled participants provided informed consent to take part in this study.

#### Overall Assessment of Risk

Participants are at minimal risk of harm associated with study participation. Although unlikely, the risks of this study are potential emotional discomfort from completing the assessments or the intervention sessions and breaches of confidentiality. In addition, participants may experience discomfort during HIV and STI testing or emotional distress when receiving positive test results. All possible steps are taken to minimize such risks through our carefully designed protocols, staff training, and fidelity monitoring. All study staff are trained in and follow study clinical protocols to protect against risks. Risks are being monitored and addressed (as outlined in the following sections) during assessments (via direct real-time triggers signaling emotional distress) and counseling sessions and in between assessment points (via communication from participants in both arms with study staff).

#### Risk of Emotional Discomfort

Participants may experience emotional distress associated with the study content. The consent document indicates that participants do not have to respond to any questions they do not wish to answer and may discontinue their participation at any time while being compensated for the portions of the study they completed.

At each assessment point, participants complete the SIDAS, with a score of ≥21 triggering an email to the project staff, who immediately alert the study counselors, who contact the participant to assess their well-being and potential need for immediate intervention in case of severe distress. All counseling resources are available to participants regardless of their ability to pay.

Should participants experience discomfort during assessments or sessions, they are contacted as described previously to assess their mental state, need for referral or immediate intervention, and capacity to continue in the study. On the basis of the counselors’ determination, the participant may continue with the study (if there is no concern about imminent harm or lack of capacity to consent) or be referred to local in-person or telehealth LGBTQ-affirmative counseling services. For any person judged to be a danger to themselves or others or in imminent need of medical or mental health services, the project staff contacts local emergency services to intervene.

#### Risk of Physical Discomfort

Biological testing for HIV, syphilis, chlamydia, and gonorrhea may be associated with physical discomfort from the finger prick or incorrect swabbing, of which participants are informed during the consent process. Information on appropriate testing procedures and risk reduction counseling is provided to all participants at the time of testing at baseline and the 12-month follow-up. Staff are available via phone and email to answer questions that participants may have about performing biological testing. These tests are routinely conducted, and therefore, potential risks are no greater than those encountered during routine medical examinations. At the start of the study, we trained 10 infectious disease physicians from the 10 study cities of București, Brașov, Timișoara, Cluj-Napoca, Iași, Constanța, Suceava, Craiova, Galați, and Satu Mare. These physicians agreed to provide confirmatory testing and treatment to participants who test positive for HIV or other STIs during the study.

#### Risk of Breach of Confidentiality

Before any assessments, all participants are assigned a study ID number. The name-ID link is kept under electronic password and firewall protection in one of the PIs’ offices at the Columbia University research space. Electronically signed consent forms are kept in a database separate from the data under password protection. Records are kept confidential, and information provided by study participants is not released to outside sources unless written consent is provided by the study participant or it is required by law (eg, suspicion of child or elder abuse and threat of imminent action on suicidal or homicidal ideation) or to protect participant well-being (eg, in the event that immediate local intervention is required following a safety assessment). The web-based intervention platform is only accessible to study staff and participants, whose log-in information is not linked to any identifying information. All participants are required, as part of the consent process, to upload to REDCap (Research Electronic Data Capture; Vanderbilt University) a photograph of their HIV and syphilis test paddle showing the results and label marked with their unique study ID. Finally, all procedures are being monitored by the Human Subjects Protection Programs at Columbia University and the study’s Data and Safety Monitoring Board (DSMB).

#### Risk Versus Benefit

Given the public health significance addressed by this first study of its kind in CEE, the social, psychological, and physical risks reviewed previously are likely to be outweighed by the new knowledge gained regarding the efficacy of this highly scalable and portable approach to reducing gay and bisexual men’s HIV transmission risk behavior and increasing their well-being. Preventing the further spread of HIV presents clear public health implications, especially in high-stigma, low-resource contexts such as Romania and other CEE countries. By participating in this study, participants may gain insight into their sexual, behavioral, and mental health that could lead to sustained behavior change. In addition, study participants may reduce their risk of acquiring HIV and STIs through involvement in our counseling or education sessions and behavioral risk tracking. Of note, participants are the first to be involved in at-home STI testing in Romania, a procedure they may adopt routinely in their lives beyond the study, both reducing their personal health risk and potentially promoting these protective practices within their networks.

#### Data Safety Monitoring Plan

Any unexpected or serious adverse events (eg, hospitalization) that occur during the course of the study will be reported by the contact PI and to the Committee on Human Research (Institutional Review Board) at Columbia University in accordance with current guidelines for reporting adverse events. The PIs meet biweekly with the team to discuss study progress and address participant safety immediately as issues arise (eg, reported suicidal ideation).

#### DSMB Function

A 5-member monitoring committee has been convened to determine safe and effective conduct and recommend the conclusion of the study if significant risks develop or if the trial is unlikely to be concluded successfully. On May 6, 2019, the team held the first DSMB meeting and has been convening annually since. The 5 members have reviewed and approved the study design and procedures and a plan for monitoring study data and interim outcomes. Starting in year 2 and annually thereafter, the study statistician has prepared and presented to the DSMB both open (pooled) and closed (stratified and with treatment arm unidentified) reports on the following data by arm: suicidality, HIV and STI test results, depression, heavy alcohol use, and HIV transmission risk behavior. Upon review of study progress, the DSMB provides a determination about study continuation to the PIs, who share it with the Institutional Review Board and National Institute of Mental Health in their annual progress report.

### Data Analyses

#### Primary and Secondary Outcomes

It is hypothesized that participants randomized to the Comunică intervention, compared to those randomized to the EAC condition, will report significantly greater decreases in the primary (HIV transmission risk behavior defined as CAS acts with HIV-positive or unknown-status partners outside the context of one’s own or one’s primary partner’s adherent PrEP use or undetectable viral load in the past 30 days) and secondary (depressive and anxiety symptoms, suicidality, heavy alcohol use, and HIV and STI testing outside of study testing) outcomes at the 4-, 8-, and 12-month follow-up visits.

#### Sample Size Justification

As we approached the end of study recruitment, three factors were considered for estimation of power: (1) while 305 participants were finally enrolled, we had estimated that we would likely only be able to recruit 288 participants by December 31, 2022 (our date of termination of recruitment), and therefore, we re-estimated power based on this number; (2) the overall participation rate for all 3 postintervention visits was 85.9% (262/305), with a high proportion of the remaining sample completing 2 postintervention assessments; (3) we had normative distributions of the study outcomes that could be used in establishing a study design and metrics for powering the primary outcome. To that end, we re-estimated the study power as described in the following paragraphs. This new estimate was approved by the DSMB and our program officer at the National Institute of Mental Health.

In the original proposal, repeated-measures mixed linear model (or generalized estimating equations [GEE]) using all 4 study visits (baseline and 4-, 8-, and 12-month follow-ups) was planned. However, the distribution of our primary outcomes (HIV transmission risk behavior) based on collected data was problematic for fitting this type of model, which is based on a central limit theorem assumption that may not manifest. Namely, there were two issues to consider: (1) at each follow-up visit, >50% of participants manifested no HIV transmission risk behavior, creating a large point mass at the lower limit of 0.2; and (2) on the other hand, the distribution of HIV transmission risk behavior acts was very skewed, with numbers of acts of >50 leading to a skewness of >3, which again contradicts normality-based methods.

Although generally accepted approaches for power estimates for this situation do not readily exist, we found one approach that will satisfy assumptions for standard normal methods and that is also amenable to conducting a power estimation. This approach is to take the average behavior over all 3 follow-up time points (4-, 8-, and 12-month assessments) as a single within-person outcome rather than evaluating the repeated measures at the 4-, 8-, and 12-month follow-ups as separate within-person outcomes. For example, if a person reported 0, 1, and 2 high-risk acts at the 4-, 8-, and 12-month follow-up visits, respectively, these would be summed together (0 + 1 + 2 = 3) and averaged over the 3 visits (3/3=1) as a single outcome of 1 high-risk sex act over the previous 30 days per follow-up period. The details of what transpired when we examined this outcome in our (still unblinded to intervention condition) data follow in the next paragraphs.

When we took the average of HIV transmission risk behavior acts of all 3 follow-up time points (ie, during the previous 4 months), only 23.9% (73/305) of participants had no HIV transmission risk behavior acts over the entire 12-month period (ie, the point mass at 0), which, given our projected sample size of 288 (or 246 evaluable participants assuming 246/288, 85.4% or a 15% loss of data as we have been observing), is low enough to treat this average as a continuous variable in a normal approximation. However, due to a few individuals reporting very high numbers of risk acts (ie, >50), this variable was still skewed (>3), so we capped (ie, Winsorized) the maximum number of average per-assessment HIV transmission risk behavior acts at 15, the upper 97th percentile of HIV transmission risk behavior. When this was done, the HIV transmission risk behavior outcome was close enough to normal (ie, skewness=1.5) for the central limit theorem to apply to the linear model presented in the following paragraph.

The power estimation approach assumes that a linear model will fit *Y* = *a* + *bX* + *cT* + ε, where *Y* is the averaged (during the previous 4 months) previous–30-day high-risk sex behavior over the 12-month behavior; *a* is the intercept; *X* is the number of baseline previous–30-day HIV transmission risk behavior acts; *T* is 0 for control and 1 for the intervention; ε is the random error with a mean of 0 and constant variance; and *a*, *b*, and *c* are unknown parameters that are estimated in the model fit. The null hypothesis *c*=0 will be tested with an overall 2-sided type-1 error of 0.05.

Importantly for power estimation, the correlation between the baseline and the average 12-month HIV transmission risk behavior acts was 0.37, which means that, after adjustment for the preintervention behavior, the SD of the postintervention behavior would be the square root of 1 – 0.37^2^ = 0.93 of the unadjusted outcome. On the basis of this, and with 288 participants, 86.1% (248/288) of whom participated in all 3 visits, as we have been observing will happen (or, conservatively, 123/288, 42.7% in each treatment arm), there is 80% power to detect an effect size of 0.33, slightly greater than our originally estimated effect size of 0.25 to 0.27 and at the upper end of the range of effect sizes found for behavioral interventions addressing multiple health outcomes among sexual minority men [84]. We believe that 0.33 represents a plausible effect size to detect in this trial given the strong distinction between the 2 intervention conditions, with one involving an active therapist-guided intervention and the other consisting of self-guided psychoeducation only. The SD of the (Winsorized) averaged outcome over 12 months of per-assessment HIV transmission risk behavior acts was 3.46 acts. Multiplying this SD by the effect size of 0.33 gives 1.14 HIV transmission risk behavior acts. This means that the study will have 80% power to detect an overall mean reduction of 1.14 HIV transmission risk behavior acts in the previous 30 days per assessment period in the intervention compared to the control condition.

It should be noted that our final analysis will most likely incorporate the partial information from men with only 1 or 2 postintervention follow-up visits through imputations, or more exactly, adjustment of the partial information for the number of follow-up periods reported; if so, this would increase power, albeit by a very modest amount.

#### Data Preparation

Skewed variables will be recoded for analytic symmetry using appropriate log, square root, or other nonlinear transformations. Should we fail to be able to find a transformation that achieves sufficient linearized normality, then robust GEE (ie, with logit or log link function) will be fitted to dichotomized or count outcomes. We will also examine variable distributions, which may suggest dichotomous and multinomial recoding relevant to our primary research questions and would increase the statistical power of our models. Dependent and independent variable values will be cross-plotted as a function of time in study and summarized using parametric and nonparametric modeling methods such as loess curves. In addition to detecting trends and temporal patterns, graphic representations of time-series data will provide knowledge of within- and between-individual variability of measurements. The results will be used to construct more complex cross-group or time analytic models.

#### Analytic Plan

The analysis of the primary and secondary outcomes will use intent to treat, with participants analyzed according to their original treatment assignment. The SAS (version 9.4; SAS Institute), SPSS (version 26.0; IBM Corp), Stata (StataCorp), and R (R Foundation for Statistical Computing) software will be used for all analyses.

#### Comparability of Treatment Groups

Differences in baseline demographic characteristics between the 2 treatment arms will be assessed using appropriate graphical and statistical methods, including summary statistics and *P* values from exact, rank, chi-square, and 2-tailed *t* tests, and ANOVAs. Of note, as the analyses progress, we will control for variables related to the study outcome in the analyses. We will also investigate whether the randomization scheme was compromised.

For the HIV transmission risk behavior primary outcome (number of CAS acts in the past 30 days with HIV-positive or unknown-status partners outside the context of one’s own or one’s primary partner’s adherent PrEP use or undetectable viral load) analyses, the statistical significance threshold for an intervention (vs control) arm effect will be a 2-sided *P* value of ≤.05. The primary outcome will be evaluated between the 2 arms at the 4-, 8-, and 12-month follow-ups combined in a repeated-measures analysis that adjusts for baseline behavior. This will be analyzed using negative binomial regression with the baseline and the 4-, 8-, and 12-month time points clustered within the same person. The main effect terms for 4, 8, and 12 months after the baseline (each time point vs baseline) will be included in the model. A single interaction term between the 4-, 8-, and 12-month measures and the intervention arm will be included in the model to test for pooled postbaseline treatment arm differences. The relative number of HIV transmission risk behavior acts with a 95% CI will quantify intervention effect. GEE with person as the cluster will be used to account for within-person repeated-measure collinearity. As a sensitivity analysis, this will be repeated including all baseline covariates that are statistically associated (*P*<.05 to enter and *P*≥.10 to leave in a stepwise selection) with HIV transmission risk behavior in negative binomial GEE models with person as the cluster and adjusting for time of visit (eg, 4, 8, and 12 months each vs baseline). If there are excess zeros at each postbaseline visit, we will consider using a zero-inflated negative binomial model instead. However, this approach will split the intervention effect parameter into 2 models and, thus, may dampen the power to detect statistical significance for an intervention that affects both parts. Thus, in this setting, we will more likely use the sensitivity analysis approach described in the following paragraph.

As a further sensitivity analysis, averaged HIV transmission risk behavior over all 3 follow-up visits (or 2 postbaseline visits if 1 visit is missing) will be used as the outcome in an analysis of covariance linear regression model that adjusts for baseline HIV transmission risk behavior as a predictor and includes treatment arm assignment as a covariate. For those who are missing 1 follow-up visit, indicator variables as to which visit is missing will be included. The mean difference in HIV transmission risk behavior acts with a 95% CI will quantify intervention effect. This will be repeated including all baseline covariates that are statistically associated (*P*<.05) with HIV transmission risk behavior in stepwise selection (*P*<.05 to enter and *P*≥.10 to leave) in the aforementioned model. Should the negative binomial model described previously fail to converge, this will become the primary analysis. The statistical significance threshold for the new (vs control) intervention arm effect will again be a 2-sided *P* value of ≤.05.

The secondary outcomes of interest (all of these assessed at baseline and at the 4-, 8-, and 12-month follow-up visits) are depression and anxiety symptoms, suicidality, and heavy alcohol use. Depression, as measured using the CES-D, will be examined as a continuous and a binary variable using a cutoff of ≥16 (indicating clinical depression). Anxiety, as measured using the BAI, will be examined as a continuous variable and using a cutoff of ≥16 (indicating potentially concerning levels of anxiety). Suicidality, as measured using the SIDAS, will be examined as a continuous variable and as a binary outcome using a cutoff of ≥21 (indicating high risk of suicidality) as well as any score >0. Heavy alcohol use, as measured using the AUDIT-C, will be examined as a continuous variable and as a binary outcome using a cutoff of ≥4. The percentage of heavy drinking days in the previous 30 days before the visit, as measured using TLFB, will be examined as a continuous variable. Due to multiple comparison issues, these will each be tested individually using a 2-sided type-1 error of 0.01 and quantified using 99% CIs. The levels of these measures at the 4-, 8-, and 12-month follow-ups will be compared (adjusting for the level at the baseline visit) between the Comunică intervention and the EAC condition in repeated-measures analyses, as described in the following paragraph.

For continuous outcomes that are heavily skewed to the right and without an excessive point mass at 0 for the 4-, 8-, and 12-month follow-ups (eg, the BAI or number of heavy drinking days), a similar approach to that described for the primary outcome analyses will be used. For continuous outcomes that are not heavily skewed to the right at the 4-, 8-, and 12-month follow-ups (eg, CES-D or AUDIT-C), repeated-measure linear regression mixed models will be fitted for outcomes at baseline and the 4-, 8-, and 12-month follow-ups with participant intercept as a fixed effect; main effects for the 4-, 8-, and 12-month follow-ups; and the single interaction term between treatment arm assignment and the time point being follow-up. In sensitivity analyses, this will be repeated including all baseline covariates that are statistically associated (*P*<.05) with the outcome in models using stepwise selection (*P*<.05 to enter and *P*≥.10 to leave). The mean postintervention difference in the outcome between the treatment arms with 99% CIs will quantify intervention effect. Binary outcomes (eg, >0 HIV transmission risk behaviors, CES-D score of ≥16, SIDAS score of ≥21, SIDAS score of ≥0, and AUDIT-C score of ≥4) will be analyzed in repeated-measures models using the baseline visit and follow-up visits at 4, 8, and 12 months. Repeated-measures GEE will be fit about individual as a cluster using a logit link function. The visit number (ie, 4, 8, and 12 months vs baseline) and treatment arm assignment will be included as main effects, as will the baseline level of the outcome being modeled. In sensitivity analyses, this will be repeated including all baseline covariates that are statistically associated (*P*<.05) with the outcome in models using stepwise selection (*P*<.05 to enter and *P*≥.10 to leave). The intervention effect will be quantified using odds ratios with 99% CIs. Finally, we will also use exact tests to compare treatment arms for having ever been diagnosed with HIV, syphilis, chlamydia, and gonorrhea during a single time point (the 12-month study follow-up).

#### Mediation Analyses

In our mediation analyses, we will examine whether changes in the proposed mediators (eg, self-efficacy for condom use or heavy alcohol use prevention, identity concealment, and internalized homophobia) precede and statistically mediate intervention effects consistent with our IMB and minority stress models. We will use path analysis or structural equation modeling to model and assess the size of the indirect effect of intervention condition on 12-month outcomes through mediators assessed at 4 and 8 months controlling for the baseline effects of these mediators.

## Results

The study was funded in September 2018, and data collection began in May 2019. The last participant follow-up was in January 2024. Currently, the data analyst is cleaning data sets for each assessment point, verifying the accuracy of scale calculations, and examining patterns of missing data in preparation for data analyses for the primary and secondary outcomes as a first step. Data analyses are scheduled to begin in April 2024. Data analysis meetings are scheduled regularly to establish timelines and examine the results as analyses are gradually being conducted. Upon completion, a list of manuscripts will be reviewed and prioritized, and the team will begin preparing them for publication.

## Discussion

This study is the first to test the efficacy of an intervention with the potential to simultaneously support the sexual (eg, HIV transmission risk behavior), behavioral (eg, heavy alcohol use), and mental (eg, depression) health of gay and bisexual men in CEE using MI support and sensitivity to the high-stigma context of the region [39,40]. This study has some potential limitations. This study is only focused on cisgender men, whereas additional groups (eg, transgender and gender-expansive individuals) may present high HIV risk and could benefit from tailored interventions. Should this intervention be efficacious, future research should consider adaptations to apply this intervention to other groups at risk of HIV and associated comorbidities, such as heavy alcohol use. In addition, this study takes place in only 1 country, potentially limiting generalizability, including to other settings where this intervention might also be beneficial. Finally, this study includes only 1 comparison condition and takes place in the context of a controlled trial rather than a real-world context suitable to test the intervention’s effectiveness. As such, pending this intervention’s efficacy confirmation in this trial, future research ought to consider expansion to other target groups and countries and implementation in real-world settings in an effectiveness trial. The resulting intervention holds promise for building a bridge from initial web-based counseling support to on-the-ground service use. If efficacious and cost-effective, the Comunică intervention presents a scalable platform to address HIV and STI risk and provide behavioral and mental health support to gay and bisexual men in other high-stigma, low-resource areas in the region (eg, Ukraine and Poland) and the United States (eg, rural areas). This study will also generate intervention content and protocols that can be replicated by international service providers who do not operate in a research capacity.

## Abbreviations

AUDIT-C: Alcohol Use Disorders Identification Test–Consumption scale
BAI: Beck Anxiety Inventory
CAS: condomless anal sex
CBST: cognitive behavioral skill training
CEE: Central and Eastern Europe
CES-D: Center for Epidemiologic Studies Depression Scale
CONSORT: Consolidated Standards of Reporting Trials
DSMB: Data and Safety Monitoring Board
EAC: education attention control
EU: European Union
GEE: generalized estimating equations
IMB: information–motivation–behavioral skills
LGBIS: Lesbian, Gay, and Bisexual Identity Scale
LGBTQ: lesbian, gay, bisexual, transgender, and queer
MI: motivational interviewing
PI: principal investigator
PrEP: pre-exposure prophylaxis
SIDAS: Suicidal Ideation Attributes Scale
STI: sexually transmitted infection
TLFB: Timeline Followback

## Data Availability

The data sets generated and analyzed during this study are available from the corresponding author on reasonable request.
